# Successful pseudopregnancy of rats by short period artificial stimulation using sonic vibration

**DOI:** 10.1038/s41598-022-05293-w

**Published:** 2022-01-24

**Authors:** Marina Endo, Shigemi Tsunoda, Hirosuke Tawara, Hisayuki Abe, Takehito Kaneko

**Affiliations:** 1 Division of Science and Engineering, Graduate School of Arts and Science, Iwate University, Morioka, Iwate 020-8551 Japan; 2 Division of Fundamental and Applied Sciences, Graduate School of Science and Engineering, Iwate University, 4-3-5 Ueda, Morioka, Iwate 020-8551 Japan; 3 Institute for Animal Reproduction, Kasumigaura, Ibaraki 300-0134 Japan

**Keywords:** Animal biotechnology, Animal physiology

## Abstract

Psuedopregnancy for embryo transfer (ET) is usually induced in rats by mating with vasectomized males. Previously, we successfully induced pseudopregnancy using sonic vibration instead (Easy-ET method). The transferred embryos developed normally. Conventionally, stimulation is performed 7 × 30 s with 5 min intervals at the day before ET. However, this protocol is time-consuming because it imitates natural mating behavior. Here, we investigated pseudopregnancy induction with shorter stimulation times. Stimulation was performed 2 × 30 s, with 30 s intervals at the proestrus stage at the day before ET. Of the transferred pronuclear or two-cell embryos, 43% or 62% developed normally, respectively. Furthermore, 67% or 68% of transferred pronuclear or two-cell embryos in rats at estrus stage stimulated on the day of ET developed normally, respectively. Pseudopregnancy was successfully induced with shorter stimulation. Furthermore, this protocol may be used to perform a single-day stimulation and ET operation at the estrus stage.

## Introduction

Reproductive technologies have been used to produce new strains and maintain genetic resources in animals^1,2^. Embryo transfer (ET) is one such essential reproductive technology, which was developed for the efficient production of laboratory and livestock animals^3–10^. ET is required for the production of genetically engineered animals, generations from cryopreserved gametes, and specific pathogen-free (SPF) animal colonies. Many genetically engineered animals, including genome edited strains, have been produced as human disease models for the analysis of gene function^11–13^. Recently, these strains have been rapidly and easily produced by the microinjection method^14–16^ and a new method which could be introduced nucleases to intact embryos using electoroporation (TAKE method) using the CRISPR-Cas system^17–19^. Cryopreservation of embryos is helpful for the maintenance of genetic resources and regulation of animal colonies^20^. Furthermore, the use of SPF animals produced by ET is vital for medical research. ET has contributed to life science research that relies on these techniques in laboratory animals^21,22^.

Female rodents require mating stimulation to maintain their pregnancy. In general, pseudopregnancy is induced in females at the proestrus stage by mating them with vasectomized males on the day before ET is performed. Although it is standard protocol for producing pseudopregnant females, it has been complicated by pairs often failing to mate, resulting in the failed induction of pseudopregnancy. Furthermore, the breeding space and costs necessary for a sufficient number of healthy females and vasectomized males may place strain on limited lab resources. In a previous study, we successfully induced pseudopregnancy in female rats by artificial stimulation using sonic vibration instead of vasectomized males (the so-called Easy-ET method)^23^. The embryos which were transferred into the oviducts of pseudopregnant female rats developed into normal offspring. In the conventional Easy-ET method, pseudopregnancy was induced in females at the proestrus stage by stimulation with sonic vibration for 30 s performed 7 times at 5 min intervals one day before ET. However, this protocol is time consuming because it is designed to imitate natural mating behavior. In this study, we studied the possibility of inducing pseudopregnancy with a shortened period of artificial stimulation using the Easy-ET method.

## Results

Table 1 shows the development of embryos transferred to rats in whom psuedopregnancy was artificially induced at the proestrus stage one day before ET. After 120 s of stimulation, 64% of two-cell embryos implanted successfully, and 48% developed into normal offspring. In the short-period stimulation, which was conducted for 30 s twice with a 30 s interval, 65% of two-cell embryos implanted, and 62% developed normally. Of pronuclear stage embryos transferred to females stimulated under the same conditions, 52% implanted and 43% developed into offspring. There were no significant differences in the development of embryos among these groups and the control group, who received 30 s stimulations 7 times at 5 min intervals or mated with vasectomised males (P > 0.05). Offspring were also obtained from morulae transferred to females who were artificially induced for 30 s twice with a 30 s interval. However, these results showed significant differences compared with the development of pronuclear and two-cell embryos and control embryos (P < 0.05).

**Table 1:** The development of embryos transferred to females who were stimulated at the proestrus stage on the day before ET.

Stimulation	Status of embryos	No. of females	No. of embryos transferred	No. (%) of embryos implanted	No. (%) of offspring
With vasectomised males	Two-cell	3	60	42 (70.0 ± 4.1)^a^	37 (61.7 ± 6.2)^c^
30 s, 7 times with 5 min interval (control)	Two-cell	3	60	38 (63.3 ± 14.3)^a^	33 (55.0 ± 17.8)^c^
120 s, 1 time with no interval	Two-cell	3	59	38 (64.3 ± 8.0)^a^	28 (47.0 ± 15.0)^c^
30 s, 2 times with 30 s interval	Two-cell	3	60	39 (65.0 ± 8.2)^a^	37 (61.7 ± 6.2)^c^
	Pronuclear	3	60	31 (51.7 ± 22.5)^a^	26 (43.3 ± 20.5)^c^
	Morula*	3	73	19 (34 ± 18.4)^b^	11 (20.7 ± 14.0)^d^

Percentages were showed as the mean ± SD.

*ET* embryo transfer.

Significant differences at P < 0.05: a vs. b; c vs. d.

*Transfer 92 h after stimulation at the proestrus stage.

Table 2 shows the development of embryos transferred to females in whom psuedopregnancy was artificially induced at the estrus stage on the morning of ET. After stimulation for 30 s twice with a 30 s interval, 75% of two-cell embryos implanted normally and 68% developed into normal offspring. These results showed no significant differences compared with the development of embryos transferred to females who were stimulated at the proestrus stage (Table 1; P > 0.05). Of the fresh pronuclear stage embryos transferred to females stimulated under the same conditions, 72% implanted successfull and 67% developed normally. These results were significantly higher compared with those of pronuclear stage embryos transferred to females who were stimulated at the proestrus stage (Table 1).

**Table 2:** The development of embryos transferred to females who were stimulated at the estrus stage on the morning of ET.

Stimulation	Status of embryos	No. of females	No. of embryos transferred	No. (%) of embryos implanted	No. (%) of offspring
30 s, 2 times, 30 s interval	Two-cell	3	60	45 (75.0 ± 4.1)	41 (68.3 ± 10.3)
	Pronuclear	3	60	43 (71.7 ± 14.3)^a^	40 (66.7 ± 14.3)^a^

Percentages were showed as the mean ± SD.

*ET* embryo transfer.

^a^Significant differences at P < 0.05, compared with females stimulated at proestrus for 30 s, 2 times with 30 s interval in Table 1.

## Discussion

In this study, we successfully induced pseudopregnancy in rats using a shortened Easy-ET method, wherein psuedopregnancy was artificially stimulated using sonic vibration. In the conventional Easy-ET method, pseudopregnancy is induced in females at the proestrus stage by sonic vibration for 30 s per stimulation 7 times in 5 min intervals on the day before ET. However, this protocol takes time because it is designed to imitate natural mating behavior. Here, we successfully induced pseudopregnancy in rats by stimulation for 30 s twice with a 30 s interval. The pronuclear and two-cell embryos transferred into the oviducts of females who underwent induced pseudopregnancy by short-period stimulation, and these embryos developed into normal offspring (Table 1).

These results show that this shorter induction method decreases the operation time for inducing pseudopregnancy by artificial stimulation. The stimulation and interval times in the conventional protocol was set based on the results obtained by monitoring copulatory behavior^23^. However, the results of this study showed that pseudopregnancy could be induced without imitating copulatory behavior. It was demonstrated that pseudopregnancy could be sufficiently induced with a shorter stimulation time than actual copulatory behavior.

Although the offspring were obtained from the morulae after transfer to females with artificially induced pseudopregnancy, their development was lower than that of pronuclear and two-cell embryos (Table 1). The reason for this is unknown from these results. The time of transplantation in morulae was obviously different from the transfer of pronuclear and two-cell embryos, because the morulae were transferred into the uterus of females at 92 h after stimulation at the proestrus stage. It is known that the sensitive interaction between the development of embryos and female conditions for embryo implantation, called the implantation window, affects the success rate of implantation^24^. Furthermore, it is thought that the uteri of mice show limited implantation capacity^25^. Further studies on optimizing the stimulation conditions for females or the culture conditions of embryos in vitro are required to improve the success rate of morula transfer.

In this study, high rates of successful development were observed in the transfer of pronuclear embryos (Table 1). In general, developmental rates were stable when two-cell embryos were transferred to females who underwent induced pseudopregnancy the day before ET. This study demonstrated that pronuclear stage embryos that were transferred on the same day as pseudopregnancy induction developed into normal offspring without significant differences. Furthermore, this study examined the development of embryos transferred to females, which induced pseudopregnancy in the morning of ET (Table 2). No significant differences were observed in the development of the offspring of two-cell embryos transferred one day before stimulation or on the same day of stimulation. Surprisingly, successful development of pronuclear stage embryos transferred to females who were induced on the morning of ET was significantly higher than that of pronuclear embryos transferred to females induced the day before ET. Genome-edited animals are generally produced by genomic modification of pronuclear stage embryos using microinjection and the TAKE method^16,19^. These results indicate that the induction of pseudopregnant females, genome editing of pronuclear stage embryos, and embryo transfer can be carried out during a one-day operation. The establishment of a one-day operation greatly contributed to the efficient production of new animal strains.

In this study, we successfully induced artificial pseudopregnancy in female rats by short-period stimulation using sonic vibration. The pronuclear and two-cell embryos that were transferred into the oviducts of these pseudopregnant female rats developed into normal offspring. This protocol is expected to be useful for the production of genome-edited animals^26–28^, production of generations from cryopreserved gametes^29,30^, and specific pathogen-free (SPF) animal colonies. Furthermore, this improved procedure could reduce the need for large breeding spaces and pain during treatment to induce pseudopregnancy and contribute to animal welfare and 3Rs.

## Methods

### Animals

Iar:Wistar-Imamichi rats (Institute for Animal Reproduction, Ibaraki, Japan) aged 9–14 weeks were used for embryo collection and subsequent embryo transfer. The breeding conditions were as follows: room temperature, 23 ± 3 °C; humidity, 50% ± 10%; and lighting from 07:00 to 19:00. All animal care and procedures performed in this study were reported in accordance with ARRIVE guidelines, and were approved by the Animal Research Committee of Iwate University and the Institute for Animal Reproduction. All methods were carried out in accordance with relevant guidelines and regulations.

### Embryo collection

Females were superovulated with an intraperitoneal injection of 300 IU/kg pregnant mare serum gonadotropin (PMSG; ASKA Animal Health Co., Ltd., Tokyo, Japan), and 300 IU/kg human chorionic gonadotropin (hCG; ASKA Animal Health Co., Ltd.) 48 h after the injection of PMSG. The females were then mated with mature males overnight. The presence of plugs confirmed the occurrence of mating. At 24 h after the injection of hCG, pronuclear stage embryos were collected by flushing the oviducts with modified Krebs–Ringer bicarbonate (mKRB) medium^31,32^. Some embryos were then cultured to the two-cell and morula stages.

### Artificial pseudopregnancy of females by easy-ET method

The estrus cycles of females were estimated by observing vaginal secretions stained with 10% Giemsa stain solution on glass slides.

**Figure 1:**
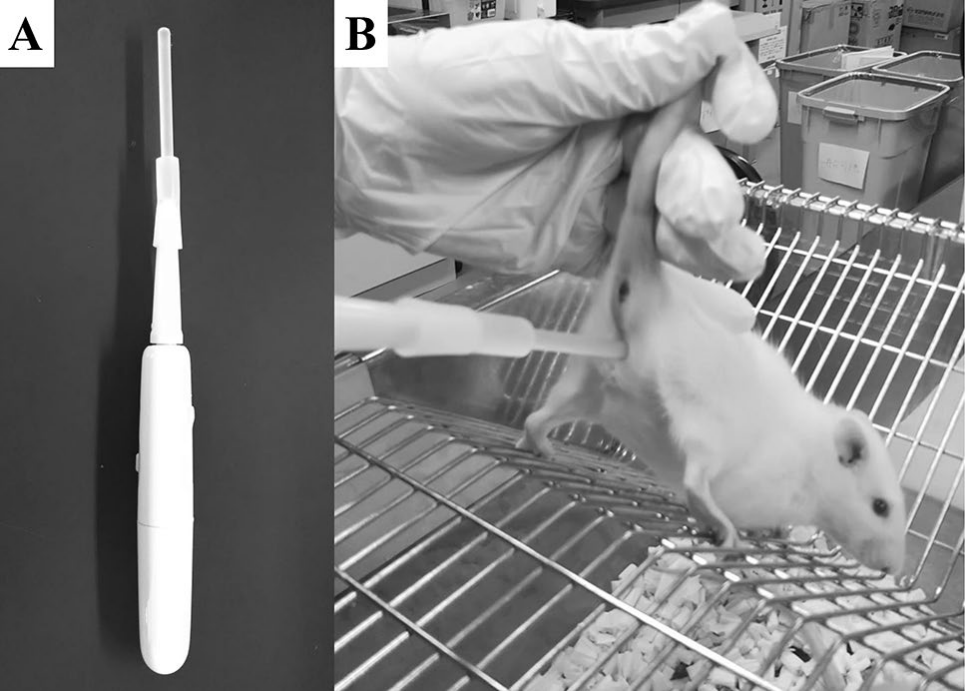
Sonic vibrator (**A**). Probe were inserted in the vagina of female (**B**).

Artificial stimulation for induction of pseudopregnancy was carried out in females at the proestrus stage at 16:00–17:00 on the day before ET. A self-made sonic vibrator (Fig. 1A)^23^ was used for artificial stimulation. The probe of the vibrator was inserted into the vagina of the female. The tip of the probe was then gently pressed into the cervical canal (Fig. 1B). Stimulation at 0.75 W power and 20,000 times per min vibration was carried out for 120 s once, or 30 s twice with 30 s intervals.

In another operation, psuedopregnancy was induced in females at the estrus stage at 9:00–10:00 on the morning of ET. These females were stimulated using the same vibrator for 30 s twice at 30 s interval. Females at the proestrus stage were stimulated for 30 s per stimulation seven times in 5 min intervals on the day before ET for control. The embryos transfer using pseudopregnant females mated with vasectomised males were also carried out as controls.

### Embryo transfer

The two-cell embryos were transferred into the oviducts of females at 17–19 h after artificial stimulation for 120 s at the proestrus stage and control. The pronuclear stage and two-cell embryos were transferred into the oviducts of females at 22–24 h and 17–19 h, respectively, after artificial stimulation for 30 s twice, at 30 s interval at the proestrus stage. The embryos that developed into morulae were transferred into the uteri of females 92 h after stimulation at the proestrus stage.

The pronuclear and two-cell embryos were transferred into the oviducts of females at 5–6 h and 1–2 h after artificial stimulation for 30 s twice with a 30 s interval at the estrus stage.

The implantation sites were observed and offspring were counted at 18 d after embryo transfer.

### Data analysis

Data were analyzed using the chi-square test followed by a multiple comparison test using Ryan’s method.

## Data Availability

The authors declare that the data supporting the findings of this study are available within the paper.
